# Biopolymer-based biomaterial containing gold nanoparticles-bioactive glass for bone regeneration in a complicated tibial fracture in a dog: a case report

**DOI:** 10.3389/fvets.2025.1568666

**Published:** 2025-09-25

**Authors:** Andreea Niculina Aștilean, Alexandra Dreancă, Ciprian Ober, Nicușor Valentin Oros, Cosmin Petru Peștean, Sorin Marian Mârza, Daniela Neagu, Klara Magyari, Cristina Gado, Liviu Oana

**Affiliations:** ^1^Faculty of Veterinary Medicine, University of Agricultural Science and Veterinary Medicine, Cluj-Napoca, Romania; ^2^Nanostructured Materials and Bio-Nano-Interfaces Center, Interdisciplinary Research Institute on Bio-Nano-Sciences, Babeș-Bolyai University, Cluj-Napoca, Romania; ^3^INSPIRE Research Platform, Babeș-Bolyai University, Cluj-Napoca, Romania

**Keywords:** tibial fractures, complications, bioactive glasses, gold nanoparticles, osteosynthesis, dog

## Abstract

A revision surgery for a tibial fracture in a dog, complicated by a secondary fracture site, implant migration, breakage, and bending of the initial implants, was described. After diaphyseal tibial osteotomy, the resulting gaps and bone defects were filled with the alginate–pullulan–bioactive glass–gold nanoparticles (Alg-Pll-BGAuSP) composite. The objective of applying this composite was to stimulate cell proliferation, based on its demonstrated bioactive effect, biodegradability, biocompatibility, and osteoinductive properties. The regenerative process was monitored both clinically and radiologically, with the aim of achieving an improved outcome and enhancing the welfare of the animal. At 12 weeks postoperatively, the implants were removed, and the limb resumed its original function. The application of this biomaterial enhanced the healing of a long bone defect, thereby promoting the formation of high-quality bone tissue in a relatively short time, despite the absence of anastomosis at the defect margins and the complexity of the case. Thus, the Alg-Pll-BGAuSP composite appears to be a viable treatment scaffold for further bone regeneration clinical trials. This first clinical report supports its potential as a scaffold for bone regeneration in complicated fractures.

## 1 Introduction

Tibial fractures are among the most common fractures in dogs, accounting for 10–20% of all cases. The majority of these fractures result from trauma, with over 50% occurring in young patients. The majority of tibial fractures are located at the diaphyseal level. Between 10 and 20% of all tibial fractures are open, and the distal region of the tibia in adult animals is most commonly affected. Immediate immobilization of the injured site is recommended following fracture occurrence (1). There is a wide range of methods for fracture repair, selection, and fixation, depending on several factors, including the type and location of the fracture, the age of the animal, the presence of defects or infection at the level of soft tissues, especially in open fractures, economic considerations, the preferences of the surgeon, and the origin of the animal (1). Usually, the fibular fracture also occurs, but its repair is not indicated if the proximal fibula or lateral malleolus is not involved (2). Along with osteosynthesis, a varied range of bioactive biomaterials can integrate into the body as biological molecules with osteoinductive and osteoconductive properties that actively support regeneration. These are considered beneficial effects and could lead to shortened healing time, especially in cases of delayed bone healing when the fracture site suffers complications (3).

Bioactive glasses are considered one of the most promising materials in the tissue regeneration process. In the *in vivo* environment, they can bond to bone via an apatite layer formation (4). By adding gold nanoparticles to the bioactive glass matrices, an enhanced material with angiogenesis properties should be obtained (5). When creating new tissues, scaffolds, cells, and bioactive cues are essential components (6). The composite of gold nanoparticles and bioactive glasses in polymeric scaffolds has attracted considerable interest due to its supportive and growth-promoting effects on surrounding cells, playing a major role in the regeneration and proliferation of injured and pathological tissues (7, 8). In our previous studies, we focused on the development of a biocompatible biomaterial composed of bioactive glass (BG) with spherical gold nanoparticles (AuSP), incorporated in the alginate–pullulan (Alg-Pll) biopolymer composite (5, 9). The results of our studies, both *in vitro* on cell cultures of fibroblasts and osteoblasts, and *in vivo* on rats, were promising, demonstrating biocompatibility, non-generation of foreign body reactions, promoting angiogenesis, and stimulating the tissues in which they were applied (5, 9, 10). Thus, these promising features led us to further evaluate the composite’s ability to induce bone healing in small animals, specifically dogs, with various pathologies or complications like those presented in this case report. Conventional bone grafting strategies, including autografts and allografts, remain the gold standard for treating critical-sized defects in veterinary orthopedics. However, autografts are limited by donor site morbidity, restricted availability, and prolonged surgical time, while allografts may carry risks of immunogenic response, disease transmission, and delayed integration. Synthetic bone substitutes, such as calcium phosphate ceramics or hydroxyapatite, provide structural support but often lack sufficient bioactivity to actively stimulate bone regeneration (11, 12). In this context, polymer-based composites incorporating bioactive glass and metallic nanoparticles have emerged as promising alternatives due to their osteoconductive, osteoinductive, and angiogenic potential. The Alg-Pll-BGAuSP composite tested in the present case report represents such an innovation, aiming to overcome the limitations of conventional grafts. To the best of our knowledge, this is the first clinical case reported of a tibial fracture caused by complications such as a delayed ossification process following the first surgery, for which this biomaterial was applied. The regeneration capacity and healing progress after the application of the Alg-Pll-BGAuSP composite were evaluated. In addition, healing time, short- and mid-term follow-up intervals, the postoperative environment assessed through clinical and radiological examinations, and restoration of the function of the right hind limb were studied. Therefore, this case report aimed to evaluate the regenerative capacity and clinical outcome of the Alg-Pll-BGAuSP composite applied in a complicated tibial fracture in a dog, with emphasis on bone healing, radiological evolution, and functional recovery.

## 2 Case presentation

**Figure 1:**
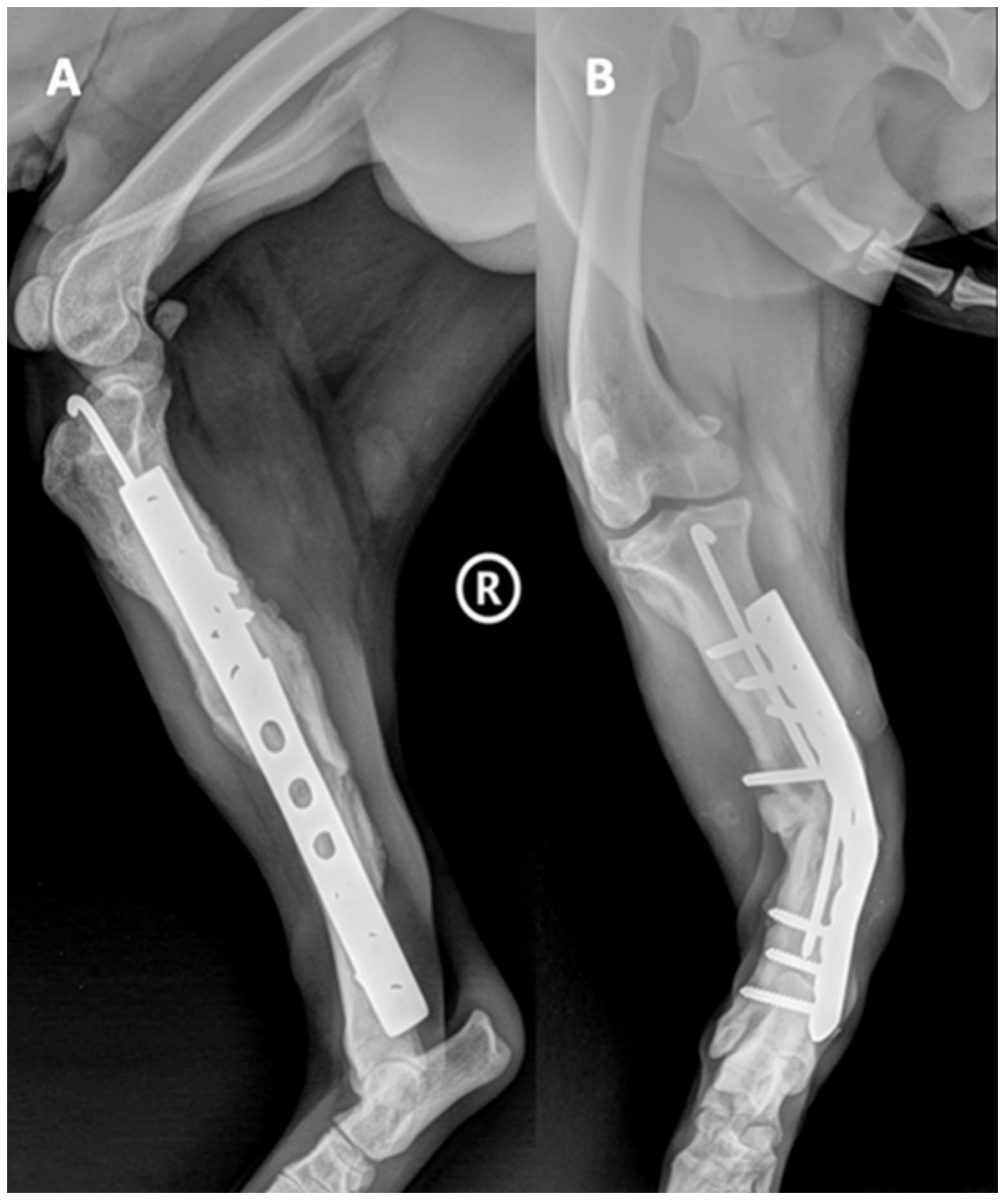
Mediolateral **(A)** and craniocaudal **(B)** radiological exposure of the right hindlimb, tibiotarsal region.

We present the case of a 3-year-old, 20-kg, mixed-breed intact male dog (BCS 5 of 9) who was referred to the Faculty of Veterinary Medicine, Cluj-Napoca, Romania, due to progressive lameness in the right hindlimb and accentuated deformity following an osteosynthesis surgery performed 4 weeks ago. After the first surgical intervention, the dog suffered a road traffic accident. He was diagnosed with an open right tibia and fibula fracture, and surgery was performed. At a surgical consultation, the dog presented a lameness score of 4 out of 5, with deformation of the limb, edema, local swelling, inflammation, heightened sensitivity, and purulent secretions. Mediolateral and craniocaudal radiographs of the right hindlimb were performed under sedation using dexmedetomidine, 0.01 mg/kg IV (Sedadex 0.5 mg/mL, Le Vet Beheer, Oudewater, Netherlands) and ketamine 3 mg/kg IV (Narkamon Bio 10%, Bioveta, Ivanovice na Hane, Czech Republic). On craniocaudal radiological exposure of the right hindlimb at the tibiotarsal region, the diaphysis of the tibia shows vicious callus, most likely result from postoperative refracture; a well-defined osteolysis zone at the middle level of the tibial diaphysis; visible orthopedic fixation elements at the level of the tibia, with a metal rod (intramedullary pin) consisting of two pieces—most likely due to the initial pin breaking into two pieces—which are correctly framed at the intramedullary level, with the tip not mobilizing the femoral-tibial-patellar joint; and the metal plate shows major unevenness due to lateral bending, while the proximal and distal locking screws remained without damage and secured the bone (Figure 1).

The animal study protocol was approved by the Ethics Committee of the University of Agricultural Science and Veterinary Medicine, Cluj-Napoca, Romania (protocol code 377 and date of approval 12.05.2023). The owner was informed about potential complications and provided consent for participation in this study through a written agreement.

## 3 Investigations

### 3.1 Composite used

For the preparation of the AuSP and BGAuSP (60SiO_2_·31.85CaO·8P_2_O_5_·0.15Au_2_O mol%), the synthesis described in the previous studies (13–15) was used. AuSPs were synthesized using the Turkevich–Frens method (16), and BGAuSPs were prepared via the sol–gel method. The gold amount introduced into the glass sample corresponded to 0.09at% in the BGAuSP. The BGAuSP was introduced in Alg-Pll biopolymer composites using a synthesis described in previous studies (5, 9, 17). The weight ratio of alginate-pullulan is 1:0.75, and the weight ratio of BGAuSP in Alg-Pll composite is 12.5 wt%. For the synthesis details and structural properties, see Supplementary Figures S1–S3.

### 3.2 Radiological examination

The case was examined using X-ray radiography at the Laboratory of Radiology and Medical Imaging, Faculty of Veterinary Medicine, Cluj-Napoca, Romania. X-ray images were obtained using a fixed X-ray apparatus, TEMCO Grx-01 (K&S Röntgenwerk Bochum GmbH&Co KG, Bochum, Germany). Exposures were made dorsoventrally, in both perspectives (lateral and medial). The parameters used to obtain the images were 60 kV and 20 mA. Images were acquired using a Rayance Xmaru 1717SGC/SCC Flat Panel Detector DR (Rayance Inc., Hwaseong-si, Gyeonggi-do, Republic of Korea) and Xmaru VetView V1 (Rayance Inc., Hwaseong-si, Gyeonggi-do, Republic of Korea) acquisition software.

### 3.3 Surgical procedures

**Figure 2:**
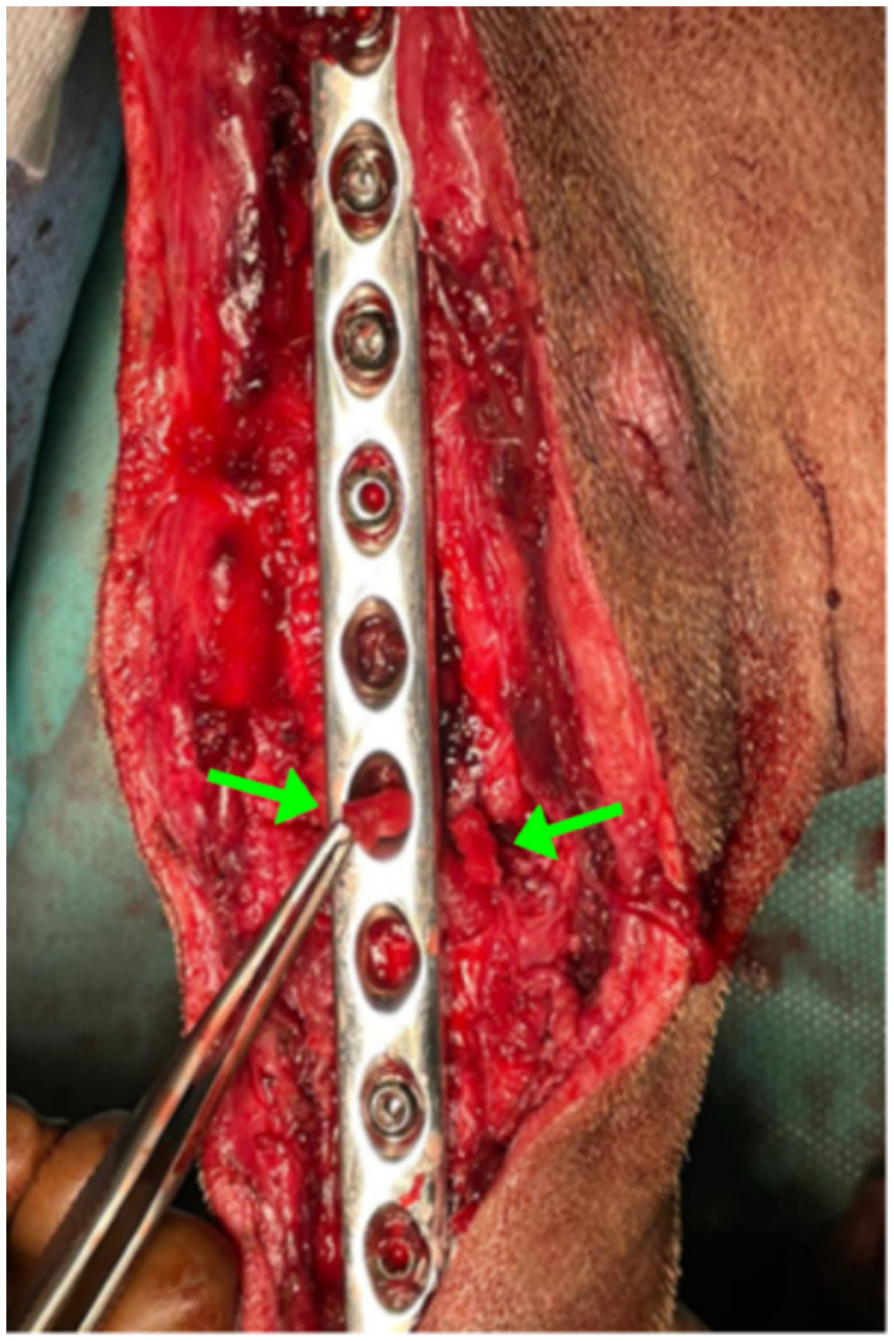
Intraoperative image showing the Alg-Pll-BGAuSP composite inserted into the fracture gap. The green arrows indicate the biomaterial within the defect site.

The surgical procedure was performed under general anesthesia. Methadone (0.2 mg/kg IM; Insistor 10 mg/mL, Richter Pharma, Wels, Austria) was administered as premedication, followed 20 min later by dexmedetomidine (0.02 mg/kg IV; Sedadex 0.5 mg/mL, Le Vet Beheer, Oudewater, Netherlands) and ketamine (2 mg/kg IV; Narkamon Bio 10%, Bioveta, Ivanovice na Hane, Czech Republic). Induction of anesthesia was performed with propofol to effect IV and maintained with isoflurane (Isoflutek 1,000 mg/g, Laboratorios Karizoo S.A., Barcelona, Spain) delivered in 70% oxygen. Cefotaxime 25 mg/kg IV (Cefotax 1 g, The Egyptian International Pharmaceutical Industries Company, Egypt) and robenacoxib 2 mg/kg SC (Onsior 20 mg, Elanco GmbH, Bad Homburg vor der Höhe, Germany) were administered 30 min before starting the surgery. Dorsal recumbency was the position, and the affected hindlimb was aseptically prepared for surgery. The shaft of the tibia was approached through a medial incision of the skin. The proximal landmarks were the medial tibial condyle, the midline of the tibia midshaft, and distally to end near the medial malleolus. The subcutis was incised on the same line. The crural fascia was incised along the cranial border of the cranial tibial muscle, starting at the tibial tuberosity and extending distally to the tendinous portion of the muscle. The implants were removed, and samples were collected for microbiological examination, including both biological tissues and parts of the implants. The area was irrigated with a sterile saline solution. At the level of the vicious callus fracture site, an osteotomy was performed with an oscillating saw (18.5 mm × 9.0 mm × 0.48 mm blade) and refracture, removal of the exuberant fibrous callus with the rongeur bone, and realignment of the bone ends. The implants used consisted of a 2.0-mm Kirschner wire intramedullary pin, a 10-hole, 3.5-mm stainless steel LC-DCP plate (limit contact-dynamic compression plate), and 7 self-tapping 3.5-mm stainless steel cortical screws to fix the plate, the length of which depends on the measurements taken intraoperatively with the help of the guide. The space of the osteotomy gap was filled with Alg-Pll-BGAuSP (Figure 2). The crural fascia was closed with 2–0 absorbable monofilament suture in a simple continuous pattern with polydioxanone. The skin was closed with 2–0 non-absorbable synthetic monofilament in a cruciate interrupted pattern with nylon.

Postoperatively, the dog was administered oral firocoxib (5 mg/kg once daily) for 7 days and doxycycline (10 mg/kg once daily) for 14 days, after the antibiogram culture. During the microbiological examination, *Staphylococcus* spp. was identified, showing sensitivity only to doxycycline among the tested antibiotics. Restriction of activities was recommended for 12 weeks. The owner was also instructed to perform passive range of motion exercises and massage of the limb. Lameness evaluation was performed two times per month, and radiographic assessment was performed at 0, 8, and 12 weeks postoperatively to evaluate implants and the healing of osteotomy. The healing was defined as the presence of bridging callus formation around the fracture site. Final healing was defined when the trabecular bone presented the mineralized callus. After healing, the implants were removed. Clinical follow-up was assessed twice per month (18), and the final radiographic follow-up was recorded 12 weeks postoperatively.

### 3.4 Treatment

This case report presents the case of a complicated fracture of a tibia and a fibula, which was treated using the Alg-Pll-BGAuSP composite and osteosynthesis. The use of this biomaterial was chosen over conventional grafting options to avoid donor-site morbidity and to test its osteoinductive potential. During the revision surgery, an osteotomy and freshening of the fracture ends were performed, resulting in a defect of 7 mm after repositioning. Because this gap exceeded cellular dimensions and direct bone-to-bone contact was not achieved, delayed or absent healing was anticipated (19). Consequently, the decision was made to use a biomaterial designed to meet the needs of such a fracture site, with the graft serving as a scaffold that provides a supportive network for cellular proliferation essential to the bone regeneration process.

### 3.5 Outcome and follow-up

Postoperative evaluations were conducted at 0, 2, 4, 6–10, and 12 weeks, including radiographic assessments, lameness scoring based on serial video recordings, clinical examinations, and bi-monthly owner check-ins. Immediately after surgery (day 0), the lameness score was 5/5, with the patient unwilling to bear weight on the affected limb. At 2 weeks, partial weight-bearing was possible (score 3/5), improving to 2/5 at 4 weeks. Between weeks 6 and 10, lameness stabilized at 1/5, and by week 12, the patient showed no lameness (0/5). A structured overview of clinical timepoints, events, and outcomes is provided in Table 1.

**Table 1:** Structured timeline of clinical, functional, and radiographic outcomes in the reported case.

Timepoint	Event	Clinical status
Day 0	Revision surgery: removal of failed implants, diaphyseal tibial osteotomy, defect filled with Alg-Pll-BGAuSP composite, and new implant fixation	Lameness 5/5 and no weight-bearing
2 weeks	Postoperative check	Partial weight-bearing and lameness 3/5
4 weeks	Follow-up	Improved limb use and lameness 2/5
6–10 weeks	Serial follow-up	Stable improvement and lameness 1/5
8 weeks	Radiographic assessment	Callus formation and defect closure tendency
12 weeks	Radiographic + clinical evaluation	Bridging callus, bony union, implants removed, and lameness 0/5
8 months	Owner video follow-up	No lameness, full function, weight gain, and no sensitivity

The table summarizes key follow-up points, including lameness score, radiographic findings, and rehabilitation protocol, allowing clear visualization of the patient’s progression from surgery to complete functional recovery.

**Figure 3:**
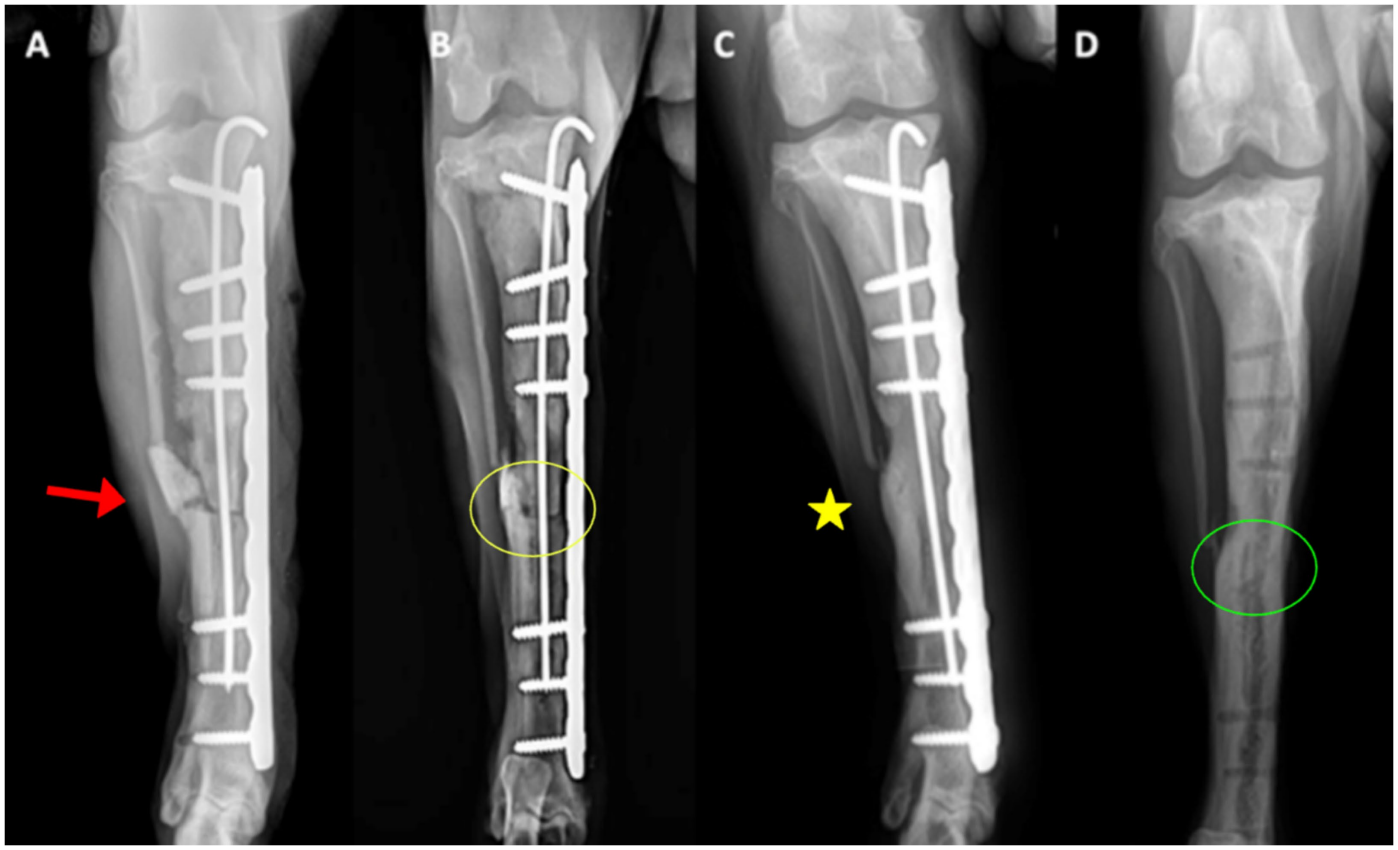
Craniocaudal radiographs of the right tibia. **(A)** Immediate postoperative view (day 0) showing correct implant positioning and alignment (red arrows). The osteotomy line is indicated by the arrowhead. **(B)** At 8 weeks, early callus formation (yellow circle) is visible around the defect, with gradual closure of the fracture gap. **(C)** At 12 weeks, the bridging callus (yellow asterisk) fully connects the fracture ends, with implants optimally positioned and no osteolysis. **(D)** After implant removal, the bone defect is completely filled with newly formed bone, restoring diaphyseal continuity (green circle).

**Figure 4:**
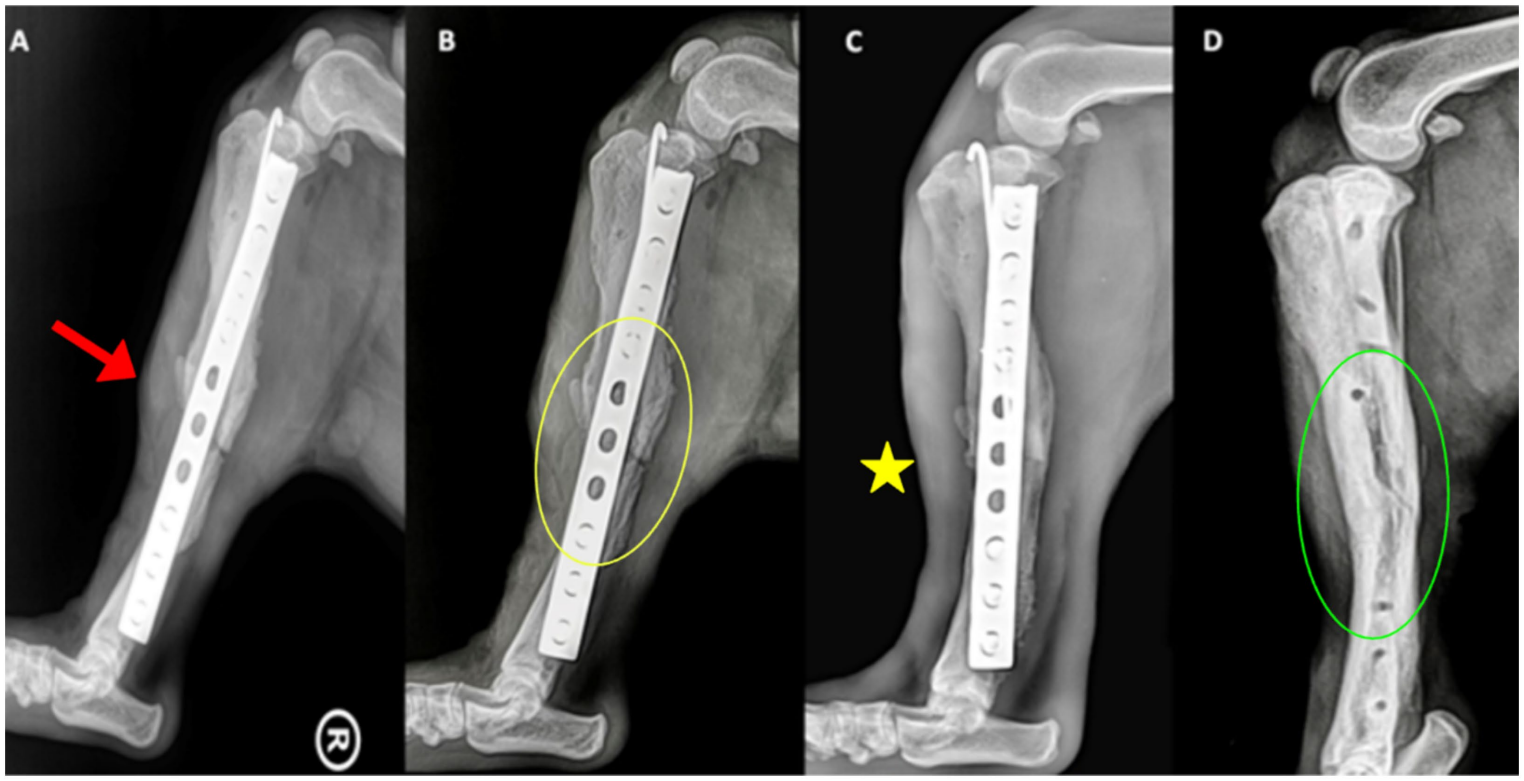
Mediolateral radiographs of the right tibia. **(A)** Day 0, showing implant alignment and osteotomy site (red arrowhead). **(B)** At 8 weeks, callus formation (yellow circle) is evident along the osteotomy margins. **(C)** At 12 weeks, the callus bridges the fracture site (yellow asterisk), with no evidence of implant loosening or inflammatory reaction. **(D)** After implant removal, the bone has fully consolidated, with smooth cortical contour restored (green circle).

Radiographs at day 0 confirmed correct implant placement and alignment (Figures 3A, 4A). At 8 weeks, bone callus formation and progressive gap closure were evident, without excessive reaction or osteolysis (Figures 3B, 4B). By week 12, the fracture site was fully bridged by a mature callus, with optimal implant positioning and no evidence of osteolysis or inflammation (Figures 3C, 4C). Consequently, implant removal was performed at week 12, with radiographs confirming complete bone healing and restoration of diaphyseal integrity (Figures 3D, 4D).

Throughout the 12 weeks, the owner adhered to the prescribed movement restrictions. Initially, the dog was walked on a leash twice daily for 10 min at a time. These walks gradually increased both in frequency and duration: at 4 weeks, three times daily for 15 min; at 8 weeks, three times daily for 15 to 20 min; and at 12 weeks, walks of 20 to 30 min were possible. The rehabilitation protocol was implemented in accordance with the orthopedic recommendations of Brinker et al. (20).

After this revision surgery, the patient recovered full function of the right hind limb, and the time of the collected data was short- and mid-time after surgery (21). Throughout the 12-week postoperative period, no adverse reactions such as local inflammation, infection, or delayed healing were observed. The owner adhered strictly to the prescribed rehabilitation protocol. At 12 weeks postoperatively, radiographic and clinical evaluations confirmed complete bone healing, and the dog was video recorded before surgery and again at 12 weeks after surgery (see Supplementary Videos S1, S2). At 8 months after the surgical intervention, medium-term follow-up via an owner-submitted video confirmed complete recovery: the patient showed no lameness, no local sensitivity, and had gained 10 kg, indicating a return to normal activity levels.

## 4 Discussion

The present case was treated according to the principles of AO/ASIF (Association for Osteosynthesis/Association for the Study of Internal Fixation) using an internal fixation method with open reduction, the removal of the previous implants, and the application of new implants (22). The correction of the deformities at the tibial level was performed through osteotomy and the removal of the old fracture site at the same time as the freshening of the bone ends. As a result, the created gap was filled with the Alg-Pll-BGAuSP composite to exert a bioactive effect on the osteoprogenitor cells, since the filling of the newly created defect and the healing and restoration of the function of the limb should be resumed as quickly as possible. According to Samoy et al. (23), functional criteria for subjective clinical outcomes are categorized as excellent (no lameness), very good (temporary, intermittent lameness in weight-bearing), fair (permanent lameness during weight-bearing), and poor (lameness without weight-bearing). In the present case report, it is noted that, during the follow-up interval, the recovery was excellent, both functionally and radiologically.

Given the complexity of the present case, it was considered relevant to compare the applied Alg-Pll-BGAuSP composite with conventional grafting options and to emphasize the physicochemical and structural properties that justify its use in critical defect repair. Conventional bone grafting strategies, including autografts and allografts, remain the clinical gold standard for treating critical-sized defects in veterinary orthopedics. Autografts provide osteogenic cells, osteoinductive cues, and an osteoconductive scaffold; however, their use is limited by donor-site morbidity, restricted volume, and increased operative time. Allografts improve availability but introduce risks of immune reactions, potential disease transmission, and typically slower host incorporation. Synthetic bone substitutes, such as calcium phosphate ceramics or hydroxyapatite, provide structural support but often lack sufficient bioactivity to actively stimulate regeneration (11, 12). In contrast, the Alg-Pll-BGAuSP composite provided an osteoconductive, highly porous scaffold with pro-angiogenic potential from gold nanoparticles within a degradable biopolymer matrix. In the present delayed-healing scenario, this translated into progressive callus formation, radiographic bridging by week 12, and complete functional recovery [lameness from 5/5 to 0/5 per Samoy et al. (23) criteria], without infection or adverse events. These outcomes align with the healing efficacy commonly pursued with autografts while avoiding donor-site complications, and they circumvent allograft-related concerns such as immunogenicity and delayed incorporation. While encouraging, these observations derive from a single case and should be interpreted as hypothesis-generating; controlled studies are required to determine whether such composites can consistently match or surpass autograft/allograft performance in delayed union or critical-defect settings (5, 9, 11, 12, 23).

A structured comparison between autografts, allografts, synthetic ceramics, and the Alg-Pll-BGAuSP composite is provided in Supplementary Table S1, highlighting their biological activity, complication rates, integration kinetics, and applicability in delayed union scenarios. The structural and morphological properties of the Alg-Pll-BGAuSP scaffold further support its clinical relevance. Its high porosity promotes cellular migration and angiogenesis (Supplementary Figure S3A). Moreover its controlled degradation kinetics (Supplementary Figure S3B) allow for progressive substitution by newly formed bone, avoiding premature collapse or persistence of non-resorbable material (5, 24). Recent research in veterinary orthopedics supports the use of polymer-based and nanoparticle-enhanced scaffolds for managing complex fractures. In a 2024 canine case report (25), combining platelet-rich plasma with hydroxyapatite nanoparticles in a severe tibial fracture showed accelerated osteogenic regeneration versus conventional care, underscoring the clinical promise of nano-augmented grafts in dogs. Complementary reviews highlight that biodegradable natural and synthetic polymer composites can be engineered for osteoconductivity and controlled degradation, enabling safe replacement by new bone in veterinary indications (26), while next-generation platforms such as shape-memory polymer scaffolds and 3D-printed constructs offer tunable mechanics and porosity aligned with canine long-bone biomechanics and defect geometry (27, 28). Within this landscape, our Alg-Pll-BGAuSP composite—combining a degradable biopolymer composite with bioactive glass and pro-angiogenic gold nanoparticles—fits a broader trend toward functionalized polymer composites designed to couple biological stimulation with temporary mechanical support (29).

Our current study is in line with a previous study performed by Ober et al. (30) in which another biomaterial based on *β*-tricalcium phosphate was used to fill the gap of a critical defect induced by the TTA (Tibial Tuberosity Advancement) rapid surgical intervention. Additionally, our study results concur well with the ones made by Lee et al. (31), in which BMP-2 protein was introduced in a non-union fracture at the radius-ulnar and tibial levels, respectively. The biomaterial was used due to several complications (failed implant and non-union fracture). Apparently, there are not many clinical case reports using biomaterials, and all of the studies have been made in recent years. It is a new trend in improving surgical strategies. Thus, the originality of our study is highlighted.

When the patient presents with phenomena of vicious or delayed bone healing, such graft biomaterials with bioactive properties are recommended and preferred by clinicians. In general, after a fracture is treated with a stabilization method, healing takes place in an average time of approximately 6 weeks when the gap is non-existent. However, if major complications occur, the healing period may extend significantly (over 12 weeks) and can vary based on the individual, surgical management, and postoperative care.

Thus, this biomaterial is recommended to be used successfully in pathological bone defects, which is why it can be a good candidate for use in cases of fractures with a lack of substance, surgical interventions involving osteotomies such as TTA Rapid, complicated fractures, or patients having precarious biological terrain with metabolic pathologies that generate slowed bone proliferation.

Although this is a case report, we can compare our results with experimental studies of various biomaterials in dogs. Extrapolating findings from *in vitro* research to the *in vivo* setting can be challenging. Because of this, testing orthopedic and dental implants on animal models is frequently a necessary step before they are used in human clinical settings (32). In this regard, the dog and sheep/goat exhibit more potential as animal models for bone implant material testing. Although no species satisfies every need for an ideal model, choosing an appropriate species for a given research issue is probably made easier with knowledge of the variations in bone architecture and remodeling among species (32). As an example, effective biomaterials for repairing critical-sized bone defects or fractures were found when biomaterials manufactured from bovine hydroxyapatite were tested in a beagle mandibular defect model (33). Similarly, another study states the safety and biocompatibility of a bovine hydroxyapatite in large mandibular bone defects (34). In other research, an intramedullary pin intended for use in the canine femur was subjected to finite element analysis. To improve their clinical utility, metallic biomaterials were compared (35).

Similar experimental models were found for bioactive glasses containing materials (36). Bioactive glasses were also tested in dogs for periodontal disease; there were experimental studies (37). In some case reports, biomaterials have been used to state that calcium phosphate ceramics are osteoinductive in the muscles of dogs during a 2.5-year study. Although the quality and quantity varied among different ceramics, the induced bone in both hydroxyapatite and *β*-tricalcium phosphate ceramics neither disappeared nor grew uncontrollably during a period as long as 2.5 years (38). Although the performance of materials in the intricate physiological environment may be understood and assessed using *in vivo* experimental animal models (39), a necessary step to clinical trials should be taken, and the results from such studies should be extrapolated into medical practice. Furthermore, a case report regarding the treatment of a non-union fracture, a 4-year-old male crossbreed dog weighing 27 kg, in overall good condition, was referred. The radiographs showed a sclerotic fracture of the end of the radius and a displacement of the ulna and radius bone fragments. Autologous adipose-derived stem cells (ADSCs) were combined with a scaffold composed of hydroxyapatite and chitosan fibers. The bone fracture was stabilized after the seeded scaffold containing ADSCs was positioned on the fracture site. The non-union fracture had healed successfully and without any issues 3 months following surgery (40). Similar to our study, using medical technology based on BMP2 materials, researchers and veterinarians from the University of Glasgow have successfully prevented the amputation of the limb of a Munsterlander dog named Eva, who is 2 years old (41). In our opinion, such clinical cases or case reports better describe real-life situations when such biomaterials are needed. Interestingly, a case report regarding the treatment of a complicated fracture with the same composite materials was not found.

One important limitation of this case report is the absence of histological or micro-CT evaluation, which would have allowed a more conclusive demonstration of scaffold integration and bone regeneration. Since the patient was a client-owned dog, invasive sampling was not ethically feasible, and micro-CT was not available in our clinical setting. Radiographic and functional assessments were therefore used as non-invasive indicators of healing. In future studies, experimental models or larger clinical cohorts should integrate histology and advanced imaging techniques to better characterize the regenerative process. Moreover, studies involving a larger dog population with follow-up periods exceeding 12 months after surgical intervention would be highly valuable. Nevertheless, our future endeavors are directed toward testing the material in TTA rapid procedures, where the surgical technique provides a more standardized model in which the regenerative process can be more clearly attributed to the biomaterial.

No adverse effects were reported during the 12-week follow-up or at the 8-month medium-term assessment, although longer-term monitoring in larger cohorts remains necessary. 12 weeks postoperatively, the dog had an excellent outcome, with the restoration of full function of the right hindlimb and the ability to walk freely. The owner was very satisfied with the outcome. After 12 weeks of movement restrictions, the patient is free of lameness. Overall, the application of the Alg-Pll-BGAuSP composite appears to be a viable treatment option in such cases.

## 5 Conclusion

In this case report, we presented the clinical application of an Alg-Pll-BGAuSP composite in the management of a complicated tibial fracture in a dog. The material demonstrated good biocompatibility, promoted callus formation, and supported complete functional recovery within 12 weeks, despite the complexity of the case and the absence of direct contact between fracture margins. Radiographic evaluations confirmed progressive bone healing, while clinical follow-up showed restoration of normal limb function without adverse reactions.

These findings suggest that Alg-Pll-BGAuSP can provide both mechanical support and biological stimulation in situations in cases of delayed bone regeneration. Although encouraging, this represents a single clinical case, and further studies involving larger cohorts and standardized models are required to confirm its consistency and therapeutic potential.

## Data Availability

The original contributions presented in the study are included in the article/Supplementary material, further inquiries can be directed to the corresponding authors.
