# Supplementary material for: Biopolymer-based biomaterial containing gold nanoparticles-bioactive glass for bone regeneration in a complicated tibial fracture in a dog: a case report
Source: Front Vet Sci. 2025 Sep 25;12:1568666. doi: 10.3389/fvets.2025.1568666 (PMC12509738; doi:10.3389/fvets.2025.1568666)
Supplement: Supplementary file 1 [file Data_Sheet_1.pdf]

Supplementary Material

1 Supplementary Data

Materials

Hydrogen tetrachloroaurate (III) hydrate ($HAuCl_4 \cdot 3H_2O$, 99.99%, Sigma-Aldrich), trisodium citrate dihydrate (ACS, 99.0%), and Pluronic F127 (powder, BioReagent, suitable for cell, Sigma-Aldrich) were used for gold nanoparticles (AuSP) synthesis.

The precursors used for the bioactive glass (BG) synthesis were tetraethyl orthosilicate (TEOS, ≥99%, Merck), triethyl phosphate (TEP, ≥99%, Merck) and calcium nitrate tetrahydrate ($Ca(NO_3)_2 \cdot 4H_2O$, ≥99%, Lach-Ner), hydrolyzed in presence of nitric acid (HNO_3, 65%).

For the synthesis of composites, sodium alginate and pullulan (from Aureobasidium pullulans) polymeric powder were obtained from Sigma-Aldrich. Cross-linking was performed with potassium chloride (KCl, 99.5%, Nordic Chemicals).

Ultrapure water and absolute ethanol were used throughout the whole experimental process. All chemicals were used as received without further purification.

Synthesis of gold nanoparticles-bioactive glass

The spherical gold nanoparticles (AuSP) were obtained using the Turkevich-Frens method (1). $HAuCl_4 \cdot 3H_2O$ solution was prepared (10^{-3} M). After this, the solution was heated to the boiling point of water. Afterward, $38.8 \cdot 10^{-3}$ M trisodium citrate solution was instantly added to the boiling gold precursor solution using a 10:1 volume ratio. The whole procedure continued for 30 min, and then it was cooled to room temperature. For further application, AuSP were stabilized using a Pluronic F127 block copolymer solution of 0.5×10^{-3} M. The solution containing the AuSP was stirred for 20 min, followed by an aging process of 24 hours to ensure the adherence of the polymer molecules. The excess of Pluronic F127 was removed by collecting the supernatant liquid from the centrifugation tubes, which were held for 30 min at 12000 rpm. The authors would also like to mention that all the stabilized AuSP were used during the preparation process of the bioactive network. Consequently, the real Au content can be considered the initial use done.

The BGAuSP ($60SiO_2 \bullet 31.85CaO \bullet 8P_2O_5 \bullet 0.15Au_2O$, mol%) sample was synthesized via the sol-gel method (2–4). The gold amount is conventionally indicated in oxidic form Au_2O. The reactants were added consecutively after 1-hour intervals, under continuous stirring. In the final step, the colloidal gold solution was added and stirred for 1 hour. The gelation was achieved in ≈2 days at 37°C, and the gels were aged for 3 days at 37°C. The maturated gels were dried at 110°C for 24 hours and thermally treated at 500°C for 2 hours. All analyses were performed on powder samples, which were milled by hand using an Agate mortar. The glass samples' granulation was similar.

Synthesis of alginate-pullulan-bioactive glass-gold nanoparticles composite

To obtain the composite polymeric solutions of 4% (w/v), the sodium alginate and pullulan powders were dissolved in 80°C ultrapure water at continuous stirring. For the uniform particle size, the

BGAuSP, was ultrasonicated. To make the sodium alginate-pullulan (Alg-Pll) mixture, a weight ratio of 1:0.75 was chosen, according to other reported studies (5,6). The BGAuSP was added to the polymeric mixture in a weight ratio of 12.5 wt%. Following mixing, the composites were cast in a 96-well plate and refrigerated at 4°C for 4 hours. The plates were frozen at -18°C for 24 hours and then lyophilized in a Vacuum Freez Dryer (BK-FD 10 Series, Biobase Bioindustry, Shandong Co., Ltd) for 24 hours. The composite materials were cross-linked in a 4% $CaCl_2$ solution for 4 hours. After the cross-linking process, the composites were lyophilized again.

Methods

The transmission electron micrograph (TEM) was recorded on FEI Technai G2 F20 high-resolution transmission electron microscope equipped with 200 kV, W cathode. The samples were suspended in H_2O and dropped on a 300 mesh Cu grid. The images obtained were interpreted using ImageJ software.

The X-ray diffraction pattern (XRD) was obtained using Shimadzu X-ray diffractometer (XRD 6000, Kyoto, Japan), operated with CuKα radiation (λ=1.54 Å) and a Ni filter. The diffraction patterns were recorded in the 2θ range 10°-80° with a scan speed of 2°/min.

Fourier Transform Infrared (FT-IR) absorption spectra were recorded in reflection configuration with a Jasco 6200 FT-IR (Jasco, Tokyo, Japan) spectrometer using the well-known KBr pellet technique, and the following parameters: room temperature, 400–4000 cm^{-1} range, 4 cm^{-1} spectral resolution.

SEM micrographs were recorded with Hitachi S-4700 Type II cold field emission scanning microscope (Tokyo, Japan) operated at an acceleration voltage of 10 kV.

The liquid displacement method was used to determine the composite porosity (6,7). Ethanol was used as a displacement liquid, and the percentage of porosity was calculated according to the following formula:

$$P(\%) = \left[\frac{W_1 - W_0}{\rho_{EtOH} \times V_0}\right] \times 100 \qquad (1)$$

where W_0 is the dry mass of the composite, W_1 is the mass of the composite saturated with ethanol, ρ_{EtOH} is the density of the ethanol, and V_0 is the initial volume of the composite scaffold, respectively.

Degradation test and swelling ratio were carried out by measuring the mass loss of the samples after different incubation days in Kokubo's simulated body fluid (8). After the incubation, the samples were rinsed with distilled water, their surface was wiped, and their wet masses (W_w) were measured. The samples were dried at 37°C for 48 hours to get their constant mass, then they were weighed to obtain their dry masses (W_d). Mass loss was calculated as a percentage according to the following formula:

$$Mass\ loss\% = \left[\frac{Wi - Wd}{Wi}\right] x100 \qquad (2)$$

where W_i is the initial mass of each sample.

Water uptake was calculated as a percentage by using the following formula:

$$Water\ uptake\% = \left[\frac{Ww - Wd}{Wd}\right] x100 \qquad (3)$$

All measurements were repeated three times.

2 Supplementary Figures

The TEM micrograph obtained by Alg-Pll-BGAuSP composite shows a small spherical AuNP with a diameter of around 25 nm (Figure S1a). The surface plasmon resonance bands of BGAuSP at 530 nm show the presence of nanoparticles with diameters around 25 nm, while the broadening of the absorption band indicates the polydispersity of gold nanoparticles in the glass structure (Figure S1b).

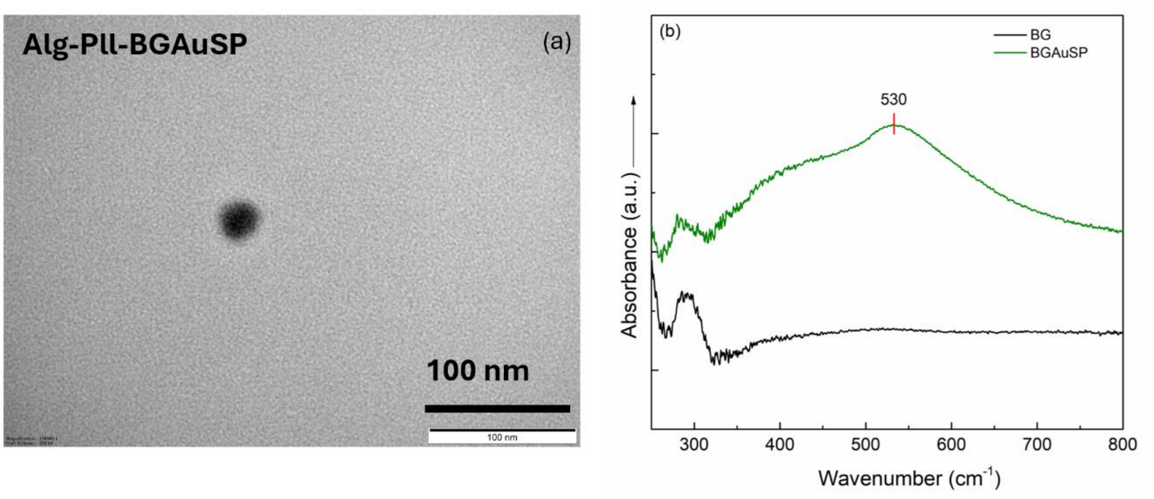

Figure S1 TEM micrograph of Alg-Pll-BGAuSP composite (a) and UV-vis spectra of BG, BGAuSP, Alg-Pll, and Alg-Pll-BGAuSP composites (b)

The XRD pattern of BGAuSP revealed the amorphous structure, whereas the presence of gold nanocrystals in the glass is proven by the reflection at $2\theta=38.1°$ (Figure S2a). The FT-IR spectra of the bioactive glasses have characteristic absorption bands of the silicate network, and the spectral characteristics are not influenced by the AuSP (Figure S2b). The XRD pattern and FT-IR spectra of bioactive glass without gold content (BG) and alginate-pullulan (Alg-Pll) composite were introduced for comparison purposes.

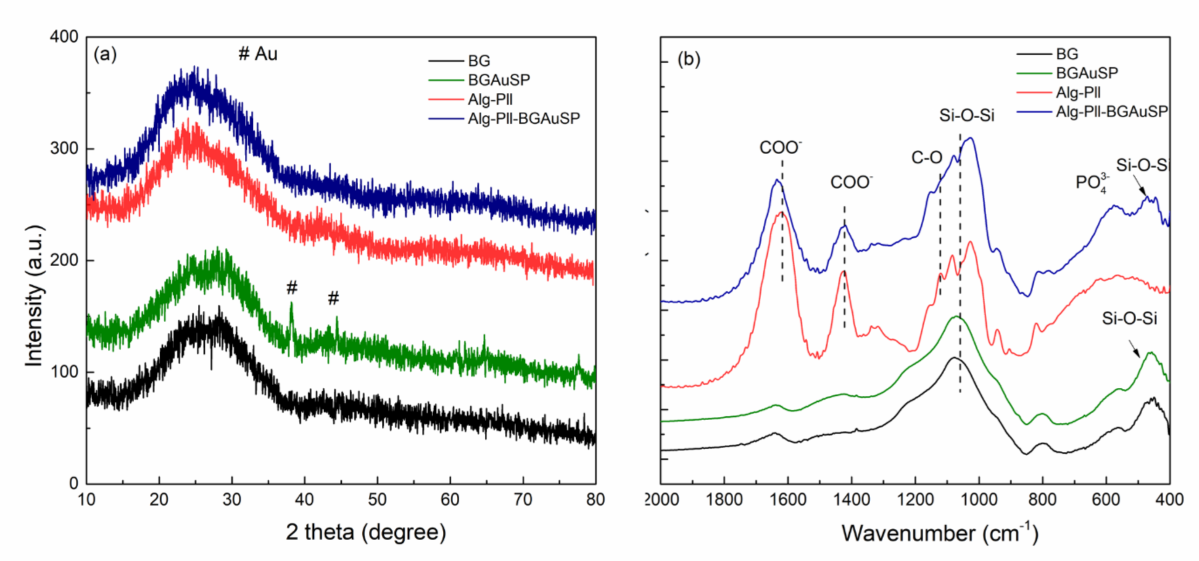

Figure S2. XRD pattern (a) and FT-IR spectra of BG, BGAuSP, Alg-Pll, and Alg-Pll-BGAuSP composites

Composite porosity was verified using the liquid displacement method (7), using ethanol as a displacement liquid, obtaining a porosity of 70%. SEM micrograph (Figure S3a) revealed that the macropores range from a few micrometers up to 100 µm, which is suitable for cell infiltration. The degradation curve shows that mass loss takes place in the first days, followed by growth due to the self-assembled apatite layer (Figure S3b). After 21 days, the composite lost 45% of the initial mass.

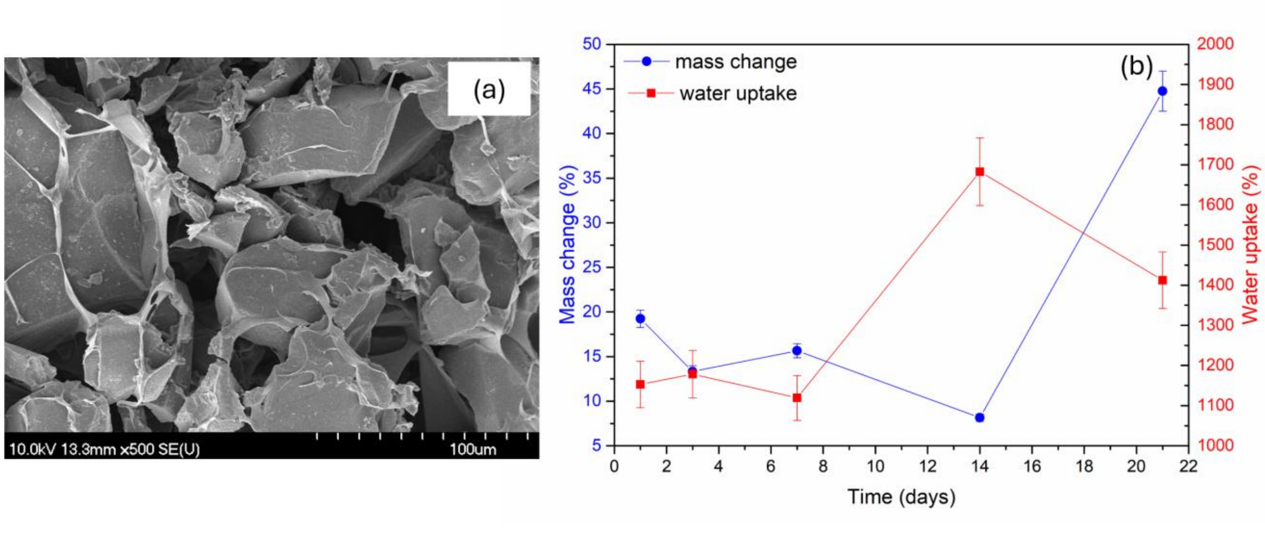

Figure S3. SEM micrograph of Alg-Pll-BGAuSP composite (a), mass change and water uptake of Alg-Pll-BGAuSP composite after different periods of immersion in simulated body fluid (b). $P<0.05$

Table S1. Side-by-side comparison of conventional grafts and the Alg-Pll-BGAuSP composite with emphasis on biological activity, complication profile, logistics, and expected integration, contextualised for delayed union.

Modality	Healing biology	Typical complications	Availability / logistics	Integration kinetics	Practical note in delayed union
Autograft (corticocancellous)	Osteogenic, osteoinductive, osteoconductive	Donor-site morbidity (pain, infection), limited volume, longer operative time	Limited by patient size and harvest site	Fastest biological incorporation	Gold standard when feasible; constraints at donor site may limit use in large defects
Allograft (cortical/cancellous)	Primarily osteoconductive (variable inductive potential)	Immunologic reaction, disease transmission (screened), slower incorporation, fracture of structural segments	Readily available, no donor site	Slower, often incomplete remodeling	Useful when autograft volume is insufficient; slower union may be suboptimal in delayed healing
Synthetic CaP ceramics (HA/β-TCP)	Osteoconductive scaffold	Brittleness, limited bioactivity, variable resorption	Off-the-shelf, scalable	Resorption and remodeling vary by formulation	Structural filler; often combined with biologics/cells to enhance healing
Alg-Pll-BGAuSP composite (this case)	Osteoconductive, pro-angiogenic (AuNP), bioactive glass surface reactivity; degradable polymer matrix	No donor-site morbidity; no immune/disease risks reported in this case; (case-level data—larger studies needed)	Off-the-shelf concept; tunable composition	Gradual resorption aligned to bone formation (case and prior in vivo data)	In this delayed-healing case, radiographic bridging and full function by week 12, with no adverse events; hypothesis-generating versus grafts

Sources: Summarised from Giannoudis et al. (9); Wang & Yeung (10); Cook et al.,(11) (reporting standards/outcomes); Samoy et al. (12) (functional criteria); Dreanca et al.,(13,14) (composite characterization and in vivo performance).

The following supporting information can be downloaded at the journal's website:

- Supplementary Video S1: Preoperative and immediate postoperative lameness assessment (day 0).
- Supplementary Video S2: Functional evaluation at 12 weeks postoperatively, showing complete recovery of limb use

Supplementary Figure S1 TEM micrograph of Alg-Pll-BGAuSP composite (a) and UV-vis spectra of BG, BGAuSP, Alg-Pll, and Alg-Pll-BGAuSP composites (b); **Figure S2.** XRD pattern (a) and FT-IR spectra of BG, BGAuSP, Alg-Pll, and Alg-Pll-BGAuSP composites; **Figure S3.** SEM micrograph of Alg-Pll-BGAuSP composite (a), mass change and water uptake of Alg-Pll-BGAuSP composite after different periods of immersion in simulated body fluid (b). $P<0.05$; **Table S1** Side-by-side comparison of conventional grafts and the Alg-Pll-BGAuSP composite with emphasis on biological activity, complication profile, logistics, and expected integration, contextualised for delayed union; **Video S1** Preoperative and immediate postoperative lameness assessment. **Video S2** Functional evaluation at 12 weeks postoperatively.

1. Frens G. Controlled Nucleation for the Regulation of the Particle Size in Monodisperse Gold Suspensions. *Nature Physical Science volume* (1973) 241:20–22.

2. Magyari K, Nagy-Simon T, Vulpoi A, Popescu RA, Licarete E, Stefan R, Hernádi K, Papuc I, Baia L. Novel bioactive glass-AuNP composites for biomedical applications. *Materials Science and Engineering C* (2017) 76: doi: 10.1016/j.msec.2017.03.138

3. Magyari K, Tóth ZR, Pap Z, Licarete E, Vodnar DC, Todea M, Gyulavári T, Hernadi K, Baia L. Insights into the effect of gold nanospheres, nanotriangles and spherical nanocages on the structural, morphological and biological properties of bioactive glasses. *J Non Cryst Solids* (2019) 522:119552. doi: 10.1016/j.jnoncrysol.2019.119552

4. Feraru A, Tóth ZR, Magyari K, Pap Z, Todea M, Mureșan-Pop M, Vodnar DC, Licarete E, Hernadi K, Baia L. Composites based on silicate bioactive glasses and silver iodide microcrystals for tissue engineering applications. *J Non Cryst Solids* (2020) 547: doi: 10.1016/j.jnoncrysol.2020.120293

5. Popescu RA, Magyari K, Taulescu M, Vulpoi A, Berce C, Bogdan S, Lelescu C, Dreancă A, Tudoran O, Papuc I, et al. New alginate–pullulan–bioactive glass composites with copper

oxide for bone tissue regeneration trials. *J Tissue Eng Regen Med* (2018) 12:2112–2121. doi: 10.1002/term.2746

6. Magyari K, Dreancă A, Székely I, Popescu A, Feraru A, Páll E, Gyulavári T, Suciu M, Cenariu M, Bobu E, et al. How does the structure of pullulan alginate composites change in the biological environment? *J Mater Sci* (2022) doi: 10.1007/s10853-022-07775-8

7. Loh QL, Choong C. Three-dimensional scaffolds for tissue engineering applications: Role of porosity and pore size. *Tissue Eng Part B Rev* (2013) 19:485–502. doi: 10.1089/ten.teb.2012.0437

8. Kokubo T, Takadama H. How useful is SBF in predicting in vivo bone bioactivity? *Biomaterials* (2006) 27:2907–2915. doi: 10.1016/j.biomaterials.2006.01.017

9. Giannoudis P V., Dinopoulos H, Tsiridis E. Bone substitutes: An update. *Injury* (2005) 36:S20–S27. doi: 10.1016/j.injury.2005.07.029

10. Wang W, Yeung KWK. Bone grafts and biomaterials substitutes for bone defect repair: A review. *Bioact Mater* (2017) 2:224–247. doi: 10.1016/j.bioactmat.2017.05.007

11. Cook JL, Evans R, Conzemius MG, Lascelles BDX, McIlwraith CW, Pozzi A, Clegg P, Innes J, Schulz K, Houlton J, et al. Proposed Definitions and Criteria for Reporting Time Frame, Outcome, and Complications For Clinical Orthopedic Studies in Veterinary Medicine. *Veterinary Surgery* (2010) 39:905–908. doi: 10.1111/j.1532-950X.2010.00763.x

12. Samoy Y, Verhoeven G, Bosmans T, Van der Vekens E, de Bakker E, Verleyen P, Van Ryssen B. TTA Rapid: Description of the Technique and Short Term Clinical Trial Results of the First 50 Cases. *Veterinary Surgery* (2015) 44:474–484. doi: 10.1111/j.1532-950X.2014.12298.x

13. Dreanca A, Muresan-Pop M, Taulescu M, Tóth ZR, Bogdan S, Pestean C, Oren S, Toma C, Popescu A, Páll E, et al. Bioactive glass-biopolymers-gold nanoparticle based composites for tissue engineering applications. *Materials Science and Engineering C* (2021) 123: doi: 10.1016/j.msec.2021.112006

14. Dreanca A, Bogdan S, Popescu A, Sand D, Pall E, Astilean AN, Pestean C, Toma C, Marza S, Taulescu M, et al. The evaluation of the osteopromoting capabilities of composites based on biopolymers and gold/silver nanoparticles doped bioactive glasses on an experimental rat bone defect. *Biomedical Materials* (2023) 18:055014. doi: 10.1088/1748-605X/ace9a6
